# Beyond the Local Basic Panel: Full CFTR Gene Analysis Identifies Novel CF Mutation Missed on Standard Testing in an Arabic Child

**DOI:** 10.7759/cureus.33337

**Published:** 2023-01-04

**Authors:** Nader Francis, Sinan Yavuz, Basil Elnazir

**Affiliations:** 1 Pediatrics/Pediatric Pulmonology, Al Qassimi Women’s & Children’s Hospital, Sharjah, ARE; 2 Pediatrics, NCH (National Children's Hospital) at Tallaght University Hospital, Dublin, IRL; 3 Pediatrics, Trinity College Dublin, Dublin, IRL

**Keywords:** cf diagnosis, cftr gene mutation, rare genetic mutation, cystic fibrosis (cf), pediatric

## Abstract

Cystic fibrosis (CF) is an autosomal recessive disease caused by different mutations in the cystic fibrosis transmembrane conductance regulator (CFTR) gene. It is the most common inherited disorder in the Caucasian population, with around 2000 mutations identified for the CFTR gene. The precise prevalence of CF in Arab countries remains unknown, with the prevalence of F508 del found to be a common type with other endemic mutations.

We describe the case of a CF patient who was diagnosed at the age of seven years. She presented post-cardiac surgery for further evaluation for a recurrent chest infection and subtle dysmorphic features. CF genetic testing for the most common 31 mutations (CF panel) was negative, and a novel mutation was identified on CFTR gene sequencing.

## Introduction

Cystic fibrosis (CF) is the most common inherited autosomal recessive disease among Caucasians. It affects approximately 1/2500 newborns in the general population, with the carrier rate of the disease ranging from 1/26 to 1/30 [1]. Around 2000 variants have been identified for the CFTR gene, and the most common mutation type is F508 del worldwide [2]. The exact prevalence of CF in Arab countries remains unknown, with the prevalence of F508 del found to be the common type with other endemic mutations [3,4]. CF is a multisystemic disease resulting in multiorgan involvement with significant morbidity and mortality due to the accumulation of thick, sticky mucus in the respiratory tract, exocrine pancreas, intestine, male genital tract, hepatobiliary system, and exocrine sweat glands [5].

Diagnosis is secured in the majority of cases by sweat testing and common CF panel mutation screen; however, novel unidentified variants can often be missed unless identified by gene sequencing. Here, we report a seven-year-old girl from the Emirati of Yemeni descent who was under assessment for dysmorphic features (triangular facies, low set ears, growth faltering, and ventricular septal defect (VSD)) with recurrent chest infection post cardiac surgery. A suspicion of CF diagnosis was confirmed by a positive sweat chloride test and phenotypical CF manifestations. The normal CF gene panel failed to identify any of the common 31 CF mutations (Elucigene Diagnostics, Manchester, United Kingdom). Extended gene sequencing identified a rare CF homozygous CFTR (NM_000492.4) variant, c.4364C>G, with a legacy name (SER1455*). Subsequent cascade screening resulted in the diagnosis of CF in another sibling.

Whole exome sequencing failed to identify any other pathogenic variant that could account for her other dysmorphic features.

## Case presentation

A seven-year-old girl, a known case of acyanotic congenital heart disease (CHD), hydrocephalus (non-communicating), dysmorphic features, and scoliosis, was referred to a pulmonology clinic for evaluation before cardiac operation. The patient was born at term as a product of first-degree consanguineous marriage. The patient’s family history is significant for a 17-year-old girl having congenital heart disease with scoliosis and another seven-year-old girl with dysmorphic features but no known family history of CF. The patient’s newborn screen was negative for elevated serum immunoreactive trypsinogen (IRT). Our patient had barium studies, which excluded congenital anomalies of the digestive tract and revealed gastroesophageal reflux and for which she underwent fundoplication. MRI brain showed moderate non-communicating hydrocephalus with tiny cavernomas. Cardiac echocardiography revealed a small atrial septal defect of 5 mm with a left to right shunt and a large VSD. She presented on two occasions, by the age of eight months, with a lower respiratory tract infection. A sweat chloride test was ordered, and it showed a concentration of 90 mmol/L and a repeated level of 100 mmol/L, which led to her diagnosis of cystic fibrosis. Subsequently, a 31 gene test -CFTR mutation panel was sent, and it was negative for CF mutations.

**Figure 1:**
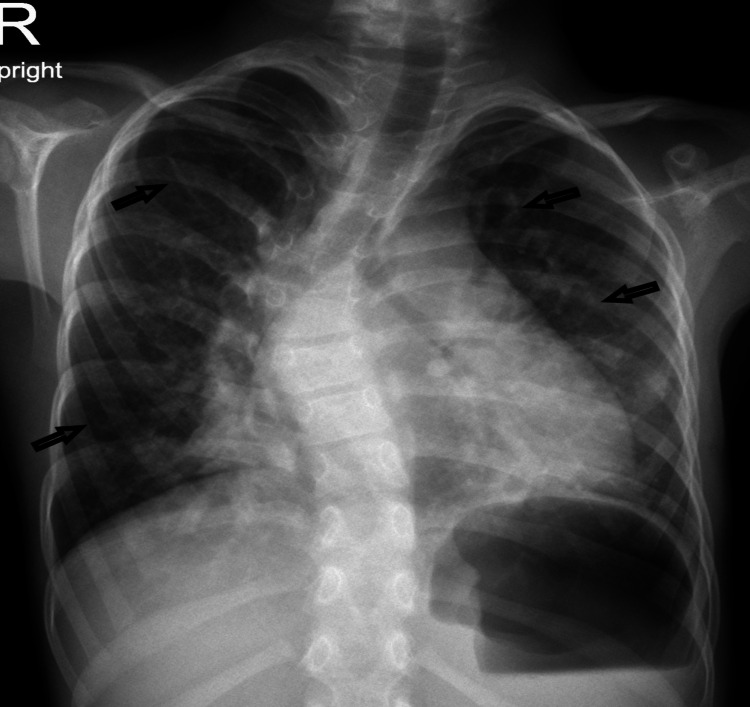
Chest X-ray showed inhomogeneous air shadowing in both lungs and marked mid-thoracic rightward scoliosis

Chest X-ray revealed inhomogeneous air shadowing in both lungs. There was marked mid-thoracic rightward scoliosis (Figure 1).

**Figure 2:**
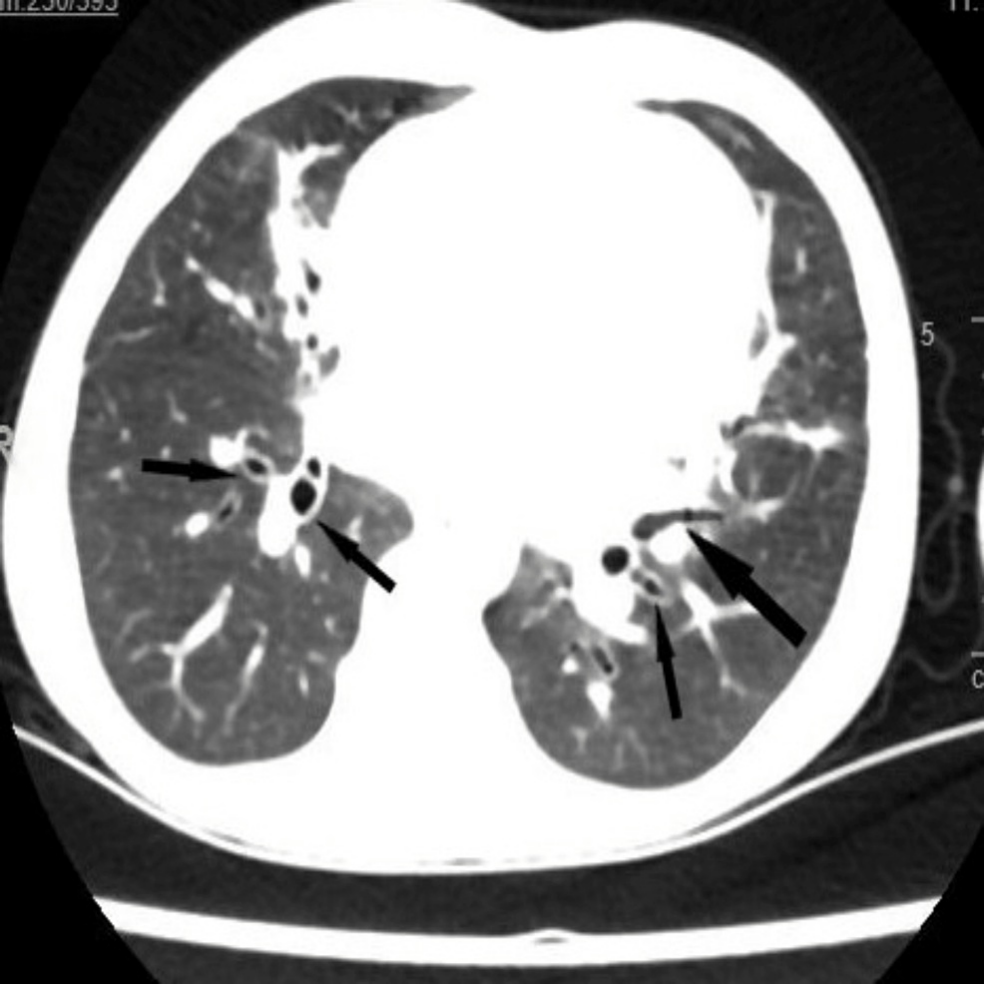
Chest CT scan showed significant tubular bronchiectasis in the upper and lower lobes bilaterally and the left lingular segments (black arrow)

The CT scan showed significant tubular bronchiectasis in the upper and lower lobes bilaterally and left lingular segments (Figure 2).

The skeletal survey showed a copper-beaten skull, bifid end first, and second right ribs, first left ribs, scoliotic deformity, thin and gracile long bones, and generalized osteopenia.

Her physical examination revealed dysmorphic features, a small fifth finger, failure to thrive, ear tag, notable chest wall deformity with scoliosis, and systolic murmur 3/6. There was no evidence of digital clubbing. She had an occasional wet cough and bilateral crackles. The rest of her systemic examination was otherwise unremarkable. She commenced an airway clearance regimen with subsequent improvement in her pulmonary status.

In view of the phenotypic manifestation and positive sweat tests, it was decided to perform extended CFTR gene sequencing. Other investigations showed normal complete blood count, electrolytes, celiac screen, immunoglobulins, minerals, vitamin A and D levels, liver function test, and iron levels. Her fecal elastase level was normal (>200 mcg/g).

Extended CFTR gene sequencing identified a novel mutation, CF apparent homozygous CFTR (NM_000492.4) variant, c.4364C>G, with legacy name p . (SER1455*), with no other mutations identified. Subsequently, a sibling was diagnosed by sweat testing and CFTR gene sequencing, which revealed the same CFTR mutation.

## Discussion

Cystic fibrosis is a genetically inherited, recessive disorder caused by mutations in the CFTR gene on chromosome 7q31. CFTR is a transmembrane chloride ion channel protein that needs adenosine triphosphate (ATP) binding and hydrolysis to function. This protein deficiency or dysfunction has systemic downstream effects on the exocrine glands, leading to elevated sweat chloride levels, thick mucus in the lungs, and severely impaired gastrointestinal absorption in 85% of patients due to exocrine pancreatic insufficiency [6,7]. CF-related diabetes, CF liver disease, and other complications usually are seen in the second decade of life. The median survival age has improved since 1986 to 39.3 years in 2014 [8].

Diagnostic tools have improved over time, permitting the exploration of rare phenotypes of CF, with some of them partially involved in CFTR gene function [9]. The CF workup algorism depends on sweat test levels [10]. Most cases of CF diagnosis are based on NBS, including or extended with CFTR gene analysis and ultimately confirmed by sweat test. But the majority of centers screen the patients with sweat tests rather than by genetic screening for the American College of Medical Genetics (ACMG) panel of the 23 most common CF-causing mutations. This panel can explore 90% of mutations in Caucasian populations, 97% in Ashkenazi Jews, and 69% in Hispanic populations [11].

The diagnosis and early established treatment before the age of two months in children have revealed better outcomes through significant improvement in respiratory and nutritional outcomes. Outcomes have been shown to be notable throughout childhood if early treatment started between the ages of four and 13 months [12].

The CFTR (NM_000492.4) variant, c.4364C>G, with the legacy name p . (SER1455*), was defined in the literature for the first time by Mickle JE et al. [13]. A comprehensive spectrum of 115 CFTR mutations in 22 Arab countries, recently published by Al-Sadeq et al., did not include the novel mutation, Ser 1455X, identified in our patient.

To our knowledge, this is the first report of such a mutation resulting in CF in the Arab region. Consanguinity is an important finding among the Arab population, with an estimated prevalence of 50% but as high as 85% among CF families [4]. In the case of our patient, her parents were first-degree relatives. Exploring novel mutations responsible for CF, especially in the Arab world will increase our diagnostic ability and result in the early implementation of appropriate therapeutic modalities. This will result in improving the median survival age, which is currently thought to be 10-20 years in the Arab population [14].

The collaborative CFTR2 project has identified 18 patients reported with this variant to the database. It identifies this variant as having varying consequences. Some patients with this variant, combined with another CF-causing variant, have CF, and other patients with this variant, combined with another CF-causing variant, do not have CF. Our patient, with consanguineous background, was homozygous for the Ser 1455 X mutation and had a positive sweat test and radiological evidence of bronchiectasis.

Our report highlights the importance of an extended gene mutation search in patients with abnormal sweat tests and the phenotypic manifestations of CF. It also reiterates the importance of reporting novel molecular epidemiological data to increase awareness about CF in the Arab world [15].

## Conclusions

CF is an autosomal, recessively transmitted, life-long disease due to mutations in the CFTR gene. More than 2000 mutations have been identified worldwide. We report a family that was found to have a rare mild phenotype of CF, which is the CFTR (NM_000492.4) variant, c.4364C>G, with the legacy name p. (SER1455*), which required extended CFTR gene sequencing for the diagnosis. It is highly recommended to extend genetic testing for CF in patients who have the phenotypic manifestations of CF disease. Early diagnosis and treatment of CF patients are essential steps in improving outcomes.
